# Supplementary material for: Surveillance of Wildlife Viruses: Insights from South Australia’s Monitoring of Rabbit Haemorrhagic Disease Virus (RHDV GI.1 and GI.2)
Source: Viruses. 2024 Sep 30;16(10):1553. doi: 10.3390/v16101553 (PMC11512407; doi:10.3390/v16101553)
Supplement: Supplementary file 1 [file viruses-16-01553-s001.zip › viruses-3220861-supplementary.pdf]

Supplementary Materials:

Molecular Methods for RHDV variant analysis

<u>Viral RNA extraction and sequencing</u>

Up to a volume of 20ml of flies of mixed species were randomly subsampled from each trap and homogenised, along with a swab of excreta from the trap walls, in an approximately equal volume of TE buffer (10mM Tris, 1mM EDTA). The swab was included to mitigate the risk of subsampled flies not including those carrying virus, because fly spots have been shown to carry RHDV [1], although Hall *et al*. [2] later found that high cross contamination between flies and fly spots in the trap makes any viral load differences between the flies insignificant. RNA was extracted from 200ul of the resulting solution using the GeneJET Viral DNA & RNA Purification Kit, following manufacturer's instructions. RNA was extracted from rabbit tissue samples using the Qiagen RNeasy minikit following manufacturer's instructions. cDNA was amplified using the Invitrogen SuperScript® III First-Strand Synthesis System. RHDV VP60 capsid protein regions were amplified from the host-virus cDNA mixture by PCR using the primers RHDVf4846, RHDVr6059, RHDVf5926 and RHDVr6986 [3], (primers detailed in Appendix 2, Supplementary Table 1) with New England Biosciences Q5® High-fidelity 2X Master Mix, using 40 PCR cycles with annealing temperature of 59°C. PCR products (a 1214 bp fragment beginning at position 4846 in the RHDV genome and a 1061 bp fragment beginning at position 5926) were visualised by agarose gel electrophoresis. Amplicons were cleaned up using 0.5X Agencourt® Ampure beads, sheared using a Covaris instrument and libraries generated with Kapa DNA library preparation reagents for 150bp paired-end sequencing on an Illumina MiSeq at the ACRF Cancer Genomics Facility (Adelaide).

<u>Sequence Filtering and Assembly</u>

Primer regions were removed from read ends using the software Cutadapt [4], discarding all reads of <35bp. Reads derived from rabbit carcasses were then aligned to RHDV reference genome NCBI NC_001543.1 using the Burrows-Wheeler Aligner BWA-MEM function. Geneious 9.1 [5] was used to call a consensus sequence for each sample using the 'highest quality' setting, trimming any ends where coverage was less than 5x.

As we were seeking to test for the spatial and temporal presence of multiple RHDV variants in fly samples, a de-novo assembly approach was employed. Reads derived from fly pools were subset into replicate sets of 15k reads for each sample using a custom script. Using the Geneious implementation of BBTools v37.28 [6], BBDuk was used to filter out read pairs with any read less than 100bp, BBMerge was used to merge paired reads with minimum overlap of 17 bases, Dedupe was used to remove duplicate reads with Kmer seed length 31bp and no substitutions permitted. BBNorm was then used to normalise reads to a target Kmer level of 50, minimum depth of 6. The resulting reads for each replicate set were de-novo assembled using MIRA 4.0 v1.1.1 [7] with accurate genome mode and no trimming. Resulting contigs were filtered to retain only those of >900bp length and consisting of at least 80 reads, for which consensuses were called using the Geneious 'highest quality' setting with minimum coverage of 6x. For each sample, contigs recovered from all replicates were pooled, deduplicted with Dedupe and then aligned to the reference sequence RHDV-V351 (GenBank accession KF594473.1). For contigs differing by <6 SNPs within each sample a single representative was retained in the alignment.

<u>RHDV Variant Analysis</u>

The software RDP4 [8] was used to screen for recombination events within each subset. This analysis used the RDP [9], GENECOV [10], MaxChi [11,12], Bootscan [13] and SiScan [14] methods. Recombination events were accepted if supported by at least three of the screening methods. Recombinant contigs were removed from the alignment prior to phylogenetic analysis.

Phylogenetic analysis was performed using RaXML as implemented in Geneious with GTR GAMMAI, rapid bootstrap+ML and 1000 bootstraps. RHDV sequence variants were assigned to clades based on phylogenetic output.

REFERENCES

1. Asgari, S.; Hardy, J.R.E.; Sinclair, R.G.; Cooke, B.D. Field evidence for mechanical transmission of rabbit haemorrhagic disease virus (RHDV) by flies (Diptera: Calliphoridae) among wild rabbits in Australia. *Virus Research* **1998**, *54*, 123-132, doi:Doi: 10.1016/s0168-1702(98)00017-3.
2. Hall, R.N.; Huang, N.; Roberts, J.; Strive, T. Carrion flies as sentinels for monitoring lagovirus activity in Australia. *Transboundary and Emerging Diseases* **2019**, *66*, 2025-2032, doi:10.1111/tbed.13250.
3. Kovaliski, J.; Sinclair, R.; Mutze, G.; Peacock, D.; Strive, T.; Abrantes, J.; Esteves, P.J.; Holmes, E.C. Molecular epidemiology of Rabbit Haemorrhagic Disease Virus in Australia: when one became many. *Molecular Ecology* **2014**, *23*, 408-420, doi:10.1111/mec.12596.
4. Martin, M. Cutadapt removes adapter sequences from high-throughput sequencing reads. *EMBnet. journal* **2011**, *17*, pp. 10-12.
5. Kearse, M.; Moir, R.; Wilson, A.; Stones-Havas, S.; Cheung, M.; Sturrock, S.; Buxton, S.; Cooper, A.; Markowitz, S.; Duran, C. Geneious Basic: an integrated and extendable desktop software platform for the organization and analysis of sequence data. *Bioinformatics* **2012**, *28*, 1647-1649.
6. Bushnell, B. *BBTools*, https://sourceforge.net/projects/bbmap/: 2017.
7. Chevreux, B.; Pfisterer, T.; Drescher, B.; Driesel, A.J.; Müller, W.E.; Wetter, T.; Suhai, S. Using the miraEST assembler for reliable and automated mRNA transcript assembly and SNP detection in sequenced ESTs. *Genome research* **2004**, *14*, 1147-1159.
8. Martin, D.P.; Murrell, B.; Golden, M.; Khoosal, A.; Muhire, B. RDP4: Detection and analysis of recombination patterns in virus genomes. *Virus evolution* **2015**, *1*, 1-5.
9. Martin, D.; Rybicki, E. RDP: detection of recombination amongst aligned sequences. *Bioinformatics* **2000**, *16*, 562-563.
10. Padidam, M.; Sawyer, S.; Fauquet, C.M. Possible emergence of new geminiviruses by frequent recombination. *Virology* **1999**, *265*, 218-225.
11. Posada, D.; Crandall, K.A. Evaluation of methods for detecting recombination from DNA sequences: computer simulations. *Proceedings of the National Academy of Sciences* **2001**, *98*, 13757-13762.
12. Smith, J.M. Analyzing the mosaic structure of genes. *Journal of molecular evolution* **1992**, *34*, 126-129.
13. Martin, D.; Posada, D.; Crandall, K.; Williamson, C. A modified bootscan algorithm for automated identification of recombinant sequences and recombination breakpoints. *AIDS Research & Human Retroviruses* **2005**, *21*, 98-102.
14. Gibbs, M.J.; Armstrong, J.S.; Gibbs, A.J. Sister-scanning: a Monte Carlo procedure for assessing signals in recombinant sequences. *Bioinformatics* **2000**, *16*, 573-582.
